# Ultra-Thin Platinum Deposits by Surface-Limited Redox Replacement of Tellurium

**DOI:** 10.3390/nano8100836

**Published:** 2018-10-15

**Authors:** Fatima Haidar, Mathieu Maas, Andrea Piarristeguy, Annie Pradel, Sara Cavaliere, Marie-Christine Record

**Affiliations:** 1 Institute Charles Gerhardt of Montpellier, UMR CNRS 5253, Chalcogenide Materials and Glasses, University of Montpellier, F-34095 Montpellier CEDEX 5, France; fatima.haidar@umontpellier.fr (F.H.); mathieu.maas@umontpellier.fr (M.M.); andrea.piarristeguy@umontpellier.fr (A.P.); annie.pradel@umontpellier.fr (A.P.); 2 Institute Charles Gerhardt of Montpellier, UMR CNRS 5253, Aggregates Interfaces and Materials for Energy, University of Montpellier, F-34095 Montpellier CEDEX 5, France; sara.cavaliere@umontpellier.fr; 3 Aix-Marseille University, University of Toulon, CNRS, IM2NP, av. Normandie-Niemen, F-13013 Marseille, France

**Keywords:** Pt thin deposits, galvanic displacement, UPD, SLRR, electrocatalysis

## Abstract

Platinum is the most employed electrocatalyst for the reactions taking place in energy converters, such as the oxygen reduction reaction in proton exchange membrane fuel cells, despite being a very low abundant element in the earth’s crust and thus extremely expensive. The search for more active electrocatalysts with ultra-low Pt loading is thus a very active field of investigation. Here, surface-limited redox replacement (SLRR) that utilizes the monolayer-limited nature of underpotential deposition (UPD) was used to prepare ultrathin deposits of Pt, using Te as sacrificial metal. Cyclic voltammetry and anodic potentiodynamic scanning experiments have been performed to determine the optimal deposition conditions. Physicochemical and electrochemical characterization of the deposited Pt was carried out. The deposit comprises a series of contiguous Pt islands that form along the grain interfaces of the Au substrate. The electrochemical surface area (ECSA) of the Pt deposit obtained after 5 replacements, estimated to be 18 m^2^/g, is in agreement with the ECSA of extended surface catalysts on flat surfaces.

## 1. Introduction

Platinum is the most employed electrocatalyst for the reactions taking place in energy converters, such as proton exchange membrane fuel cells, in particular for the oxygen reduction reaction (ORR) taking place at the cathode side [1,2,3]. To enhance its surface area and reactivity, it is often used in the form of nanoparticles. Nevertheless, a more effective utilization of platinum is crucial, due to its low abundance in the earth’s crust and its high price [4]. Despite their advantages and proven electroactivity, the search for more active electrocatalysts with ultra-low Pt loading highlights several limitations of Pt nanoparticles [5]. Indeed, their ORR activity is related to particle size and the type of exposed crystal planes [6]. Moreover, the limited interface between discrete nanoparticles and the support means there is a tendency towards migration and agglomeration during fuel cell operations, with a consequent loss of performance [7]. The development of nanostructured thin films [8] with excellent activity and stability demonstrates the potential of Pt extended surfaces. Their conformal morphology minimizes unused platinum, allowing for high ORR-specific activity [9] while preventing electrochemical Ostwald ripening [10]. The preparation of Pt thin films to achieve ultra-low Pt loaded electrodes with high performance and stability is a challenge however, due to the complexity of several fabrication techniques and the difficulty in tailoring thickness and continuity of the deposits [11,12,13]. For this purpose, electrochemical fabrication methods are often considered, due to their ease of use and overall cost effectiveness [14,15,16]. Conventional overpotential electrodeposition results in preferential growth at areas with high surface energy, such as step edges and surface defects, leading to incomplete substrate coverage at low thicknesses and non-uniform deposits [17,18]. Surface-limited redox replacement (SLRR) utilizes the monolayer-limited nature of underpotential deposition (UPD) to form an atomic layer of a sacrificial metal, coupled with the galvanic displacement of this sacrificial metal by the deposited metal [19]. This can be repeated over multiple cycles in order to allow for the growth of films of desired thickness [20]. To date, SLRR has been utilized to deposit ultrathin conformal films of Pt using Pb and Cu [21,22,23,24,25] as sacrificial metals. This led to incomplete deposition due to the different oxidation state of the metal ions (two copper (II) or lead (II) exchanged for one platinum (IV)). In this regard, tellurium can be a valuable alternative, as already demonstrated, for the formation of Pt nanostructures further applied in electrocatalysis [26,27,28], since, based on the stoichiometric relationship of the galvanic replacement reaction (Equation (1)), equivalent molar amounts of Pt atoms are generated while Te ions dissolve in the solution.

Pt(IV)Cl_6_^2−^ + Te(0) + 3H_2_O → Pt(0) + Te(IV)O_3_^2−^ + 6Cl^−^ + 6H^+^ (1)

The goal of this work was to investigate the deposition of Pt films on a model surface, such as a flat gold substrate, by SLRR, using tellurium as sacrificial metal. After having optimized and maximized the tellurium deposition and the Pt replacement, the Pt layers obtained after various numbers of cycles were characterized by scanning electron microscopy (SEM), coupled with energy dispersive X-ray spectroscopy (EDS), atomic force microscopy (AFM), inductively coupled plasma-mass spectrometry (ICP-MS), and cyclic voltammetry. 

## 2. Materials and Methods 

### 2.1. Solutions and Chemicals

All chemicals were of analytical grade and were used as received. Aqueous solutions were prepared using ACS reagents or higher-grade chemicals (Sigma Aldrich, Inc., St. Louis, MO, USA) and ultrapure water (Milli-Q > 18.2 MΩ cm, Merck KGaA, Darmstadt, Germany). The tellurium oxide solution was prepared with TeO_2_ (99.999%) and HClO_4_ as supporting electrolytes, with concentrations of 10^−3^ M and 0.4 M, respectively. Hexachloroplatinic acid hexahydrate H_2_PtCl_6_ 6H_2_O (ACS reagent, ≥ 37.50% Pt basis), 10^−3^ M, and HClO_4_ 0.1 M were used as reagent and supporting electrolytes, respectively. The supporting electrolyte HClO_4_ 0.1 M was also used as a blank solution. The pH of these solutions was 1.0. Before each series of measurements, solutions were freshly prepared, magnetically stirred, and thoroughly out-gassed with high purity nitrogen gas (N_2_) for 30 min, in order to flush out dissolved oxygen. 

### 2.2. Electrochemical Studies

All electrochemical experiments were performed using a Solartron analytical Modulab workstation and carried out using an electrochemical flow cell with three electrodes. An Ag/AgCl (3 M NaCl) served as the reference electrode (AMETEK, Inc., Berwyn, PA, USA) and a platinum wire (Sigma Aldrich, Inc., St. Louis, MO, USA) was used as the counter electrode. The working electrode was a gold rotating disk electrode (RDE) (PINE instruments, Grove City, PA, USA). For the ICP-MS analysis, a melted gold working electrode had been prepared and kept inside a resin, and the electrical connection was assured with a copper wire. For the SEM-EDS and AFM measurements, gold substrates (PHASIS, Inc., Plan-les-Ouates, Geneva, Switzerland) consisting of quartz slides coated with 200 nm thick gold films (99.9%, pure), were used as working electrodes. Under the Au film, a thin (10 nm) Ti under-layer was deposited to improve the adhesion of gold onto quartz. 

All electrochemical measurements were performed at room temperature (≈ 25 °C). All the potentials in this paper are referring to the reversible hydrogen electrode (RHE). Pt electrochemical surface area (ECSA) was evaluated using the hydrogen underpotential deposition region by integrating the charge from 0.05 to 0.4 V vs. RHE, after the subtraction of the double layer capacitance and assuming a charge density of 210 μC/cm^2^ for polycrystalline Pt [29].

#### 2.2.1. Preparation of the Gold Electrode

Before the deposition, the gold electrode was polished mechanically with diamond powder (1 µm, 0.3 µm and 0.05 µm), then ultrasonicated in water for 5 min while the gold substrate was pretreated as follows: it was first annealed at 350 °C for 18 h at 10^−6^ Torr in sealed glass tubes and then soaked in hot nitric acid for 5 min. 

The electrodes were then treated electrochemically using cyclic voltammetry (CV) in a blank solution. To clean the working electrode, 40 cycles from −0.30 V to 1.65 V vs. RHE with a scan rate of 100 mV/s were performed until a reproducible voltammogram was obtained (Figure S1, Supporting Information, SI).

#### 2.2.2. Underpotential Deposition (UPD) of Te and Te Replacement by Pt

The electrochemical behavior of Te on bare gold and on platinum electrodes was studied from a solution of TeO_2_ (Sigma Aldrich 99.99% purity, Inc., St. Louis, MO, USA) 1 mM in HClO_4_ 0.1 M, using cyclic voltammetry with various potential windows. The underpotential deposition (UPD) was carried out in the potentiostatic mode. Following the Te underpotential deposition on a gold electrode, the Te covered electrodes were rinsed in a blank solution of 0.1 M HClO_4_ and dipped into a 1 mM H_2_PtCl_6_ 6H_2_O + 0.1 M HClO_4_ solution at open circuit voltage (OCV), in order to form a Pt monolayer via surface-limited redox replacement of tellurium. The Te deposition and its replacement by Pt were progressively optimized, using results from cyclic voltammetry and anodic potentiodynamic scanning. 

### 2.3. Characterization of the Pt Deposits

Several methods were used to provide evidence for and to quantify the presence of Pt on the electrodes. First, X-ray diffraction (XRD) patterns after 5 cycles of Te UPD and Pt replacement were recorded in a Bragg-Brentano configuration using a PANalytical X’Pert diffractometer (PANalytical, Almelo, The Netherlands). The deposited layers were then observed using (i) an Oxford X-Max 50mm^2^ instrument (Oxford Instruments, Abingdon, Oxfordshire, England) equipped with energy-dispersive X-ray spectroscopy analysis (EDS), (ii) and a Bruker Nanoscope Dimension 3100 atomic force microscope (AFM, Bruker, Palaiseau, France). The AFM experiments were performed in the tapping mode under ambient conditions. The cantilever tips were Silicon Point Probe Plus^®^ NCSTR (Nanosensors, Neuchâtel, Switzerland; force constant 6.5 N/m, resonance frequency 157 kHz). All the image treatments were performed using Gwyddion [Czech Metrology Institute, Brno, Czech Republic, open software, http://gwyddion.net/]. Inductively coupled plasma-mass spectrometry (ICP-MS) analysis was used to quantify the amount of platinum deposited. In this case, the deposited films were dissolved in 10 mL of *aqua regia* and the obtained solution was analyzed. In order to obtain a sufficient amount of Pt for ICP-MS analyses, several cycles (3 and 5) of Te UPD and Pt replacement were performed.

Finally, cyclic voltammetry in nitrogen saturated HClO_4_ 0.1 M was carried out at a scan rate of 40 mV/s, to observe hydrogen UPD on Pt and evaluate from it its electrochemical surface area (ECSA). 

## 3. Results and Discussion

### 3.1. Underpotential Deposition of Tellurium on Gold

**Figure 1:**
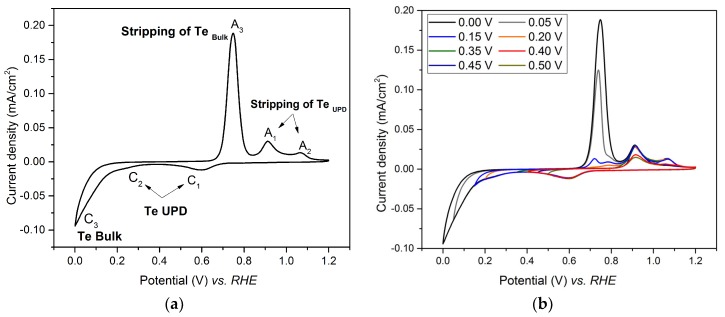
Cyclic voltammetry (CV) of the gold substrate in a solution of 0.4 M HClO_4_ and 1 mM TeO_2_ (pH 1.0) at a potential sweep rate of 40 mV/s. (**a**) CV obtained by scanning the potential from 1.20 V to 0.00V vs. RHE; (**b**) CV obtained by scanning the potential from 1.20 V to progressively lower potential limits (0.50 V, 0.45 V, 0.40 V, 0.35 V, 0.20 V, 0.15 V, 0.05 V and 0.00 V vs. RHE).

Shown in Figure 1 is the cyclic voltammogram (CV) of the Au electrode immersed in tellurium oxide solution, obtained after several consecutive voltammetry cycles, successively scanned from 1.2 V to various potential limits in the range 0–1.2 V vs. RHE at a scan rate of 40 mV/s. The shape of the CV is comparable to that recorded during underpotential deposition of Te on gold in sulfuric acid solutions by Suggs and Stickney [30].

The reduction of TeO_2_ started at 0.7 V vs. RHE (peak C_1_), in agreement with previous work attributing this reduction to the first tellurium UPD peak [31,32,33,34]. The second peak (C_2_), occurring at the lower potential of 0.4 V vs. RHE, has also been already reported [32,33] and is referred to as the second tellurium UPD, which is only partially resolved from the bulk reduction peak. It is known that the UPD peak shape and potential strongly depend on the crystallographic plane on which it occurs [35,36,37]. This can explain the slight shift between the UPD potential observed in this work, and those reported in the literature [38]. 

Detailed CV analysis was performed to identify the location of the two UPD peaks and the peak corresponding to bulk deposition by progressively extending the scanned potential region. The cathodic potential was scanned from 1.2 V vs. RHE to various lower limit values (between 0.50 V and 0.00 V vs. RHE), from which the anodic curves were recorded. The corresponding CVs are displayed in Figure 1b.

A reduction peak C1 and an oxidation peak A1 are seen when the potential was scanned between 1.2 V and 0.5 V vs. RHE. When the potential limit decreases, the intensity of the A1 peak increases and reaches its maximum for a limit value of 0.45 V. This observation suggests that C1 corresponds to a UPD peak for Te, and A1 to its corresponding stripping peak [39]. When the limiting value of the potential further decreases, an additional oxidative peak, A2, is observed. Its intensity increases when the potential limit of the scan decreases, and reaches its maximum when the limit potential is 0.35 V. This suggests the presence of a second UPD peak, C2. When the limit potential value further decreases, an additional reductive peak, C3, appears as well as its corresponding oxidative peak, A3. The intensity of the A1 peak increases as the limit potential value decreases, while the intensity of the peaks A2 and A3 remains constant. The peak C3 should be related to the bulk deposition. From these observations we conclude that the total UPD of Te on gold is reached by using a deposition potential of 0.35 V vs. RHE.

The linear sweep voltammetry (LSV) after UPD Te deposition (after polarization at 0.35 V vs. RHE) was carried out for different durations (Figure S2, Supporting Information, SI). The deposition time was thus optimized from these experiments, and 240 s was found to be the minimum time required to achieve a complete UPD. Beyond this duration, the stripping curves overlaid, indicating that the maximum intensity of the stripping peak had been reached. 

The [I-t] curve (Figure S3, Supporting Information, SI) recorded during the deposition at 0.35 V vs. RHE shows that the signal stabilized after 240 s, which is in agreement with the LSV study.

In order to obtain the maximum amount of Te by UPD, and compare with the values reported above (0.35 V and 240 s), a longer deposition duration (300 s) was adopted. 

A longer deposition duration (300 s) was adopted in order to obtain the maximum amount of deposited tellurium by UPD. In order to quantify the deposition in these conditions (0.35 V and 300 s), the deposited layer was stripped by applying a potential at 0.35 V and, using the electric charge related to the gold reduction (cf. Supplementary Information), the coverage of the electrode is estimated to be 0.87 monolayers (ML) for total UPD (C_1_ and C_2_) [36]. This demonstrates that the UPD deposition was successful and that an almost complete film of tellurium on the gold surface was obtained.

### 3.2. Investigation of Te UPD on Platinum Electrode

The aim here was to perform a multilayer deposition of platinum (by galvanic displacement) onto tellurium deposited by UPD. The possibility of further depositing tellurium onto the formed platinum layer and continuing the successive cycles of UPD/galvanic displacement on it needs to be assessed. For that, a study of Te UPD on a Pt flat electrode was performed and discussed. The same experimental method was used as was for the Au working electrode, but using a Pt electrode instead.

Before the deposition, the Pt electrode was polished mechanically and then treated electrochemically using cyclic voltammetry in a blank solution of 0.1 M perchloric acid. To clean the electrode surface, 40 cycles from −0.05 V to 1.4 V vs. RHE with a scan rate of 100 mV/s were performed until a reproducible voltammogram was obtained (Figure S4, SI).

**Figure 2:**
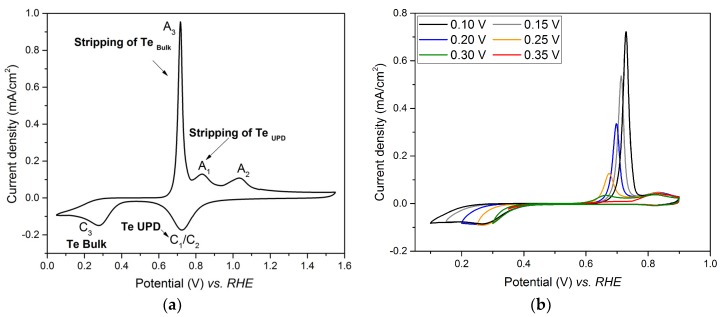
Cyclic voltammograms performed on Pt electrode in 1 mM TeO_2_ + 0.4 M HClO_4_ solution with a scan rate of 40 mV/s. (**a**) potential comprised between 1.60 V and 0.05 V; (**b**) potential from 0.9 V to various cathodic potential limits (0.35 V, 0.30 V, 0.25 V, 0.25 V, 0.15 V and 0.10 V).

Figure 2a shows cyclic voltammograms performed on Pt electrode in 1 mM of TeO_2_ with a scan rate of 40 mV/s. While C_1_/C_2_ corresponds to the tellurium UPD, and the reduction of platinum oxide to Pt, and A_1_ and A_2_ corresponds to their stripping peaks, C_3_ corresponds to the bulk reduction of Te and A_3_ to its stripping peak.

**Figure 3:**
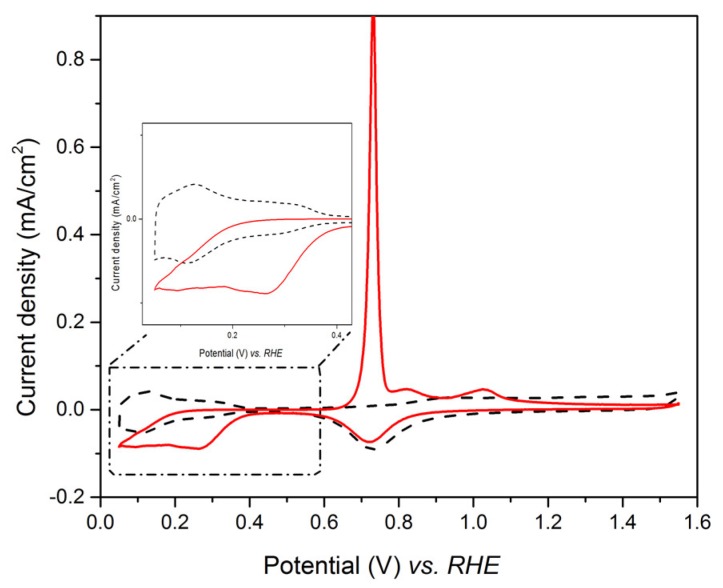
Steady-state voltammetric response of the Pt electrode in 0.1 M HClO_4_ (dotted line) and first cycle profile after Pt electrode polarization in 1 mM TeO_2_ for 450 s at 0.35 V vs. RHE (full line), sweep rate = 40 mV/s.

Since an overlap occurs between the Te UPD peak, C_1_/C_2_, and the peak of platinum oxide reduction, the investigation of the Te UPD imposed to avoid high anodic potential (such as 1.6 V vs. RHE as in Figure 2a), which led to the formation of the oxidized species. This can be achieved by limiting the upper potential range to 0.9V vs. RHE, where the platinum oxidation does not occur (Figure 2b). Indeed, when the potential is scanned from 0.9 V to 0.35 V vs. RHE, only A_1_ appears on the anodic curve. When the potential limit is below 0.35 V, the bulk A_3_ peak appears. Thus, the underpotential deposition of tellurium on platinum has to be performed between 0.35 V and 0.30 V vs. RHE. The experimental conditions for Te under potential deposition were chosen as E = 0.35 V and t = 450 s. The voltammogram recorded in the range 0.05 V–1.55 V vs. RHE after Te UPD at 0.35 V for 450 s is shown in Figure 3 (full line plot) and compared to that obtained for the Pt electrode in 0.1 M HClO_4_ (dotted line). The tellurium deposit totally inhibits H adsorption on the Pt surface, as observed in the potential range between 0.40 and 0.05 V vs. RHE.

Using the electric charge related to the platinum reduction, the Te coverage of the electrode is estimated to be 0.86 ML [40]. The UPD deposition of Te on Pt is thus effective.

### 3.3. Te Replacement by Pt

**Figure 4:**
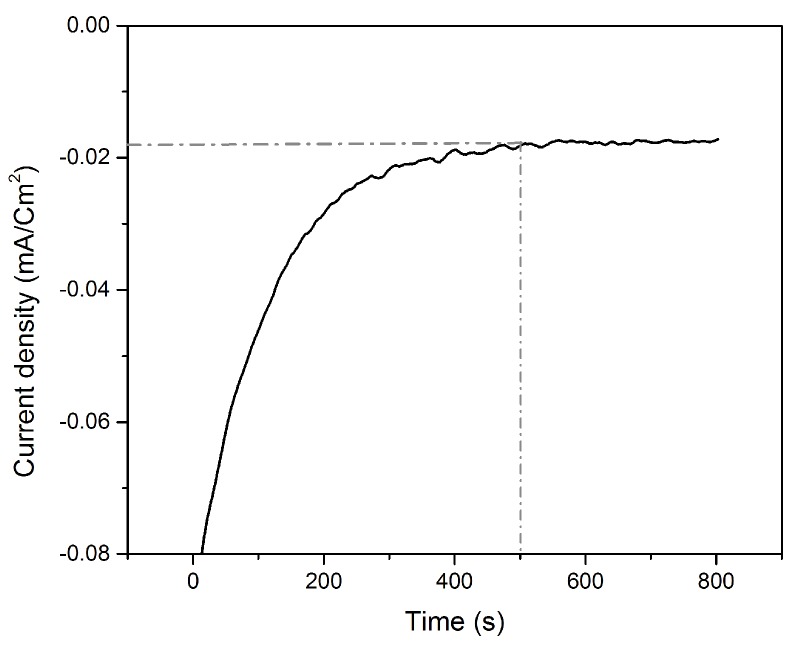
Chronoamperometric curve of the galvanic replacement of Te by Pt at open circuit voltage (OCV).

Following the Te underpotential deposition on bare gold, the Te film was galvanically exchanged with platinum via SLRR. The atomic layer of Te was then oxidized while PtCl_6_^2−^ ions were reduced, replacing the Te layer by a Pt layer. The time needed to obtain the highest replacement rate was been determined from the I-t curve (Figure 4), and is 450 s.

### 3.4. Underpotential Deposition of Te onto Pt-Covered Gold Electrode and Subsequent Te Replacements by Pt

**Figure 5:**
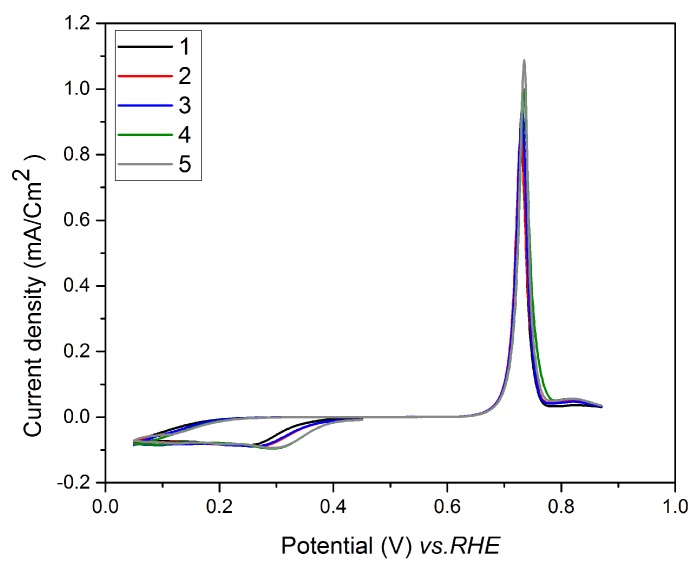
Cyclic voltammograms on Pt-covered Au electrode in 1 mM TeO_2_ with a scan rate of 40 mV/s (after the number of replacements indicated in the figure).

The voltammograms obtained on a Pt-covered gold electrode immersed in Te solution after various numbers of replacements (from 1 to 5) are presented in Figure 5. The Au electrode was scanned from 0.45 V to 0.05 V vs. RHE and then up to 0.87 V vs. RHE only, in order to avoid the oxidation of the Pt layer obtained by the previous replacements. One can observe that the intensity of the Te UPD peak increases with the number of replacements and that its potential (0.35 V vs. RHE) is similar to that determined on a platinum bulk electrode (Section 3.2). This latter result is not surprising, since the electrical resistivity of the Pt layer should be very low.

**Figure 6:**
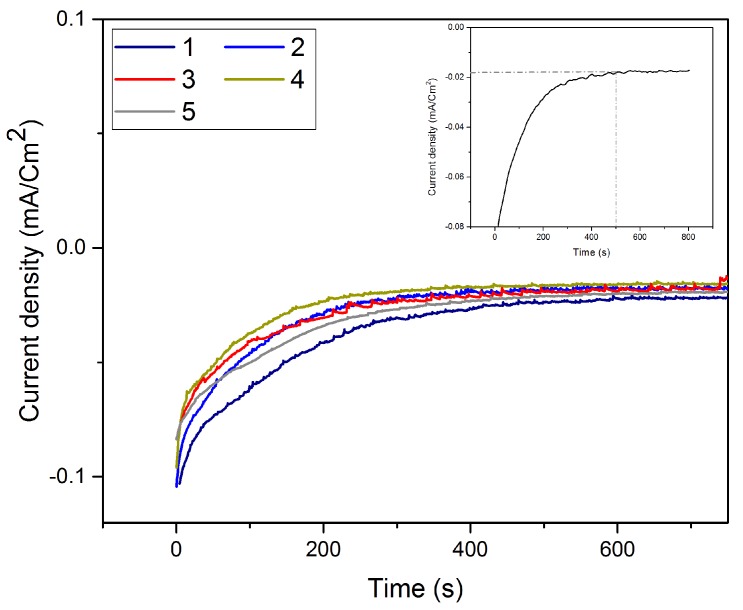
I-t curves recorded at OCV during the Pt replacement of Te layer for various numbers of replacements.

The I-t curves (Figure 6) recorded during the successive replacements of Te with Pt show that irrespective of the number of replacements, the signal stabilized after 450 s. All these results allow us to define the following experimental conditions: E = 0.35 V and t = 300 s for the Te underpotential deposition; E = OCV and t = 450 s for the Pt replacement.

### 3.5. Characterization of the Pt Film

**Figure 7:**
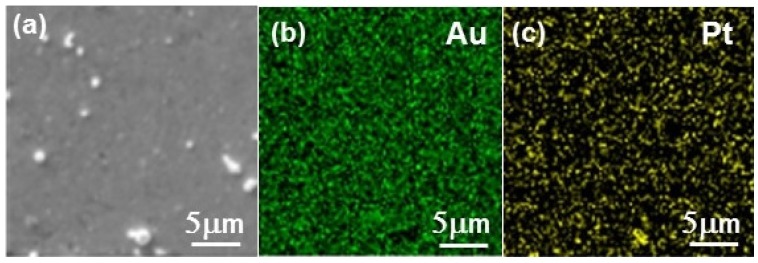
SEM image (**a**) and energy dispersive X-ray spectroscopy (EDS) mapping showing (**b**) Au and (**c**) Pt chemical contrasts for the Pt layer deposited on the gold-coated glass slide after 5 cycles of Te deposition and Pt replacement.

**Figure 8:**
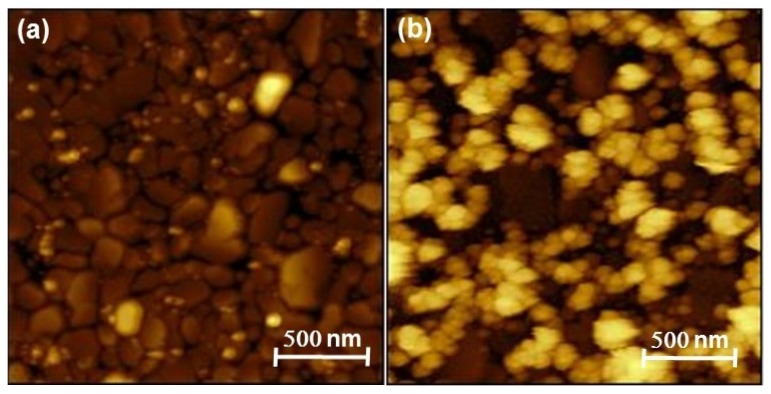
Atomic force microscopy (AFM) micrographs of (**a**) a bare Au-coated glass slide and (**b**) Pt deposit on Au-coated glass slide.

The success of the Pt deposition was difficult to assess, due to (i) the ultra-low amount formed on the electrode, which was extremely difficult to characterize, (ii) the impossibility of observing the Pt deposit on the gold rotating disk electrode (RDE) directly. RDE was replaced by a 200 nm gold coated quartz slide in a series of experiments of Te deposition and Pt replacement. The Au/quartz slides were then cut in small pieces that could fit the chambers of the different characterization instruments. No information could be obtained from X-ray diffraction, in agreement with the low amount of Pt involved (diffractograms not shown here). Figure 7 shows the SEM image and EDS mappings of the layer after 5 cycles of Te deposition and Pt replacement. They show the presence of Pt distributed over the whole surface of the support. No Te could be detected, which demonstrates its effective replacement by Pt (see the EDS spectra in Figure S5, Supporting Information, SI). The morphology of the layer analyzed by AFM is shown in Figure 8, along with the morphology of the substrate. The latter comprises gold grains of different shapes and dimensions. While the substrate is not completely covered, it appears that the Pt tends to deposit at the gold grain interfaces easier than on the grain surface, leading to a series of contiguous platinum islands formed along the Au-grain interfaces.

The quantity of deposited Pt has been determined by ICP-MS analysis after 3 and 5 replacement cycles. Taking into account the concentration given by the analyses (0.696 ppb and 3.339 ppb for 3 and 5 cycles, respectively), we conclude that 0.20 ML of Pt were deposited onto the electrode for 3 Te replacements and 0.52 ML for five ones. No trace of tellurium was detected in the analyzed solutions. This allows us to believe that the deposited tellurium has been replaced. 

**Figure 9:**
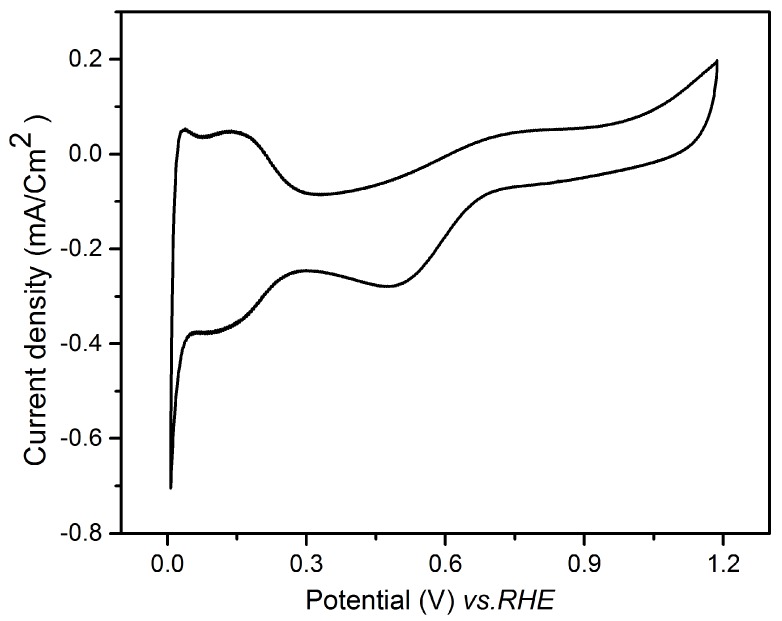
Cyclic voltammogram recorded in 0.1 M HClO_4_ on Au/Te electrode after five galvanic replacements with Pt.

Figure 9 presents a cyclic voltammogram recorded in the range 0.05–1.2 V vs. RHE after five galvanic replacements on the Au electrode. The CV demonstrated the successful deposition of Pt, exhibiting the three characteristic regions of this metal: the hydrogen adsorption/desorption peaks in the potential range between 0.05–0.35 V vs. RHE, the double layer capacitance, and the Pt oxide formation with the corresponding reduction [41]. It should be noticed that the CV presents a certain distortion and asymmetry, probably due to resistive effects, because of the ultra-low amount of Pt and thus difficult electrical connection of the electrode.

The electrochemical surface area (ECSA) of the Pt film obtained after 5 replacements was estimated to be 18 m^2^/g. This value is in agreement with the ECSA of extended surface catalysts on flat surfaces that in general is lower than that of the corresponding nanoparticles, but gives rise to higher specific activities [42]. 

The aim of the present work was to investigate the feasibility of ultra-thin Pt deposits by successive cycles of tellurium underpotential deposition and its subsequent replacement with platinum by galvanic displacement. To date, up to five cycles have been achieved, which allowed us to demonstrate the successful replacement of Te by platinum. While the coverage is not complete, platinum deposits along the Au-substrate grains in the form of contiguous islands, a feature that is looked for, since sintering should be energetically unfavourable and the oxide-dissolution mechanism leading to Pt loss should also be reduced. The low ECSA argues for flat Pt islands, again a feature that is looked for, since it should give rise to enhanced specific activities.

On the whole, these first results are encouraging and pave the way to the deposition of ultra-thin Pt layers for utilization as catalysts in electrochemical energy conversion devices. To reach the final goal of a high mass activity, combining high specific activity (presence of active sites) and high ECSA (presence of available surface), the deposition of thin films here reported for a model surface will be extended to nanostructured supports. The mechanical properties such the elastic modulus and the residual stress will then be studied, since they might affect the adhesion of the deposit on the substrate, a major concern when dealing with flexible supports or even free-standing films [43,44].

## 4. Conclusions

In this work, we studied the deposition of Pt atomic layers onto gold substrate. The deposits were performed by successive cycles, each one consisting in Te underpotential deposition and subsequent Pt replacement of Te by galvanic displacement. The optimal conditions for the underpotential deposition of Te (E = 0.35 V and t = 300 s) on gold and platinum electrodes as well as those for the Pt replacement of Te (OCV for 450 ms) were determined using cyclic voltammetry and anodic potentiodynamic scanning.

The deposited Pt was characterized by scanning electron microscopy coupled with energy dispersive X-ray spectroscopy, atomic force microscopy, inductively coupled plasma-mass spectrometry and cyclic voltammetry. It demonstrated a deposition from 0.2 to 0.5 monolayers of the noble metal, in the form of Pt contiguous islands that form at the substrate Au-grain interfaces preferentially.

The surface-limited redox replacement applied in this work on a model flat gold surface demonstrated to be an effective and straightforward method to obtain platinum ultra-thin deposits. The next step will be its application on electrocatalyst supports used in proton exchange membrane fuel cells, to further validate the possibility of tuning the thickness and ensuring a conformal morphology on more complex structures.

## Supplementary Materials

The following are available online at http://www.mdpi.com/2079-4991/8/10/836/s1. Figure S1: Cyclic voltammogram of the Au electrode in 0.1 M HClO4 at a scan rate of 100 mV/s, Figure S2: Linear sweep voltammetry after tellurium UPD deposition for various times of deposition at 0.35 V vs. RHE. (a) peak A1; (b) peak A2, Figure S3: Cyclic voltammogram of the Pt electrode in 0.1 M HClO4 at a scan rate of 100 mV/s, Figure S4: Cyclic voltammogram of the Pt electrode in 0.1 M HClO4 at a scan rate of 100 mV/s, Figure S5: EDS spectra of (a) bare Au-coated glass slide and (b) Pt deposit on Au-coated glass slide. Red arrows indicate the expected position of Te peaks, showing the absence of the element in the layer.
