# Supplementary material for: Ultra-Thin Platinum Deposits by Surface-Limited Redox Replacement of Tellurium
Source: Nanomaterials (Basel). 2018 Oct 15;8(10):836. doi: 10.3390/nano8100836 (PMC6215156; doi:10.3390/nano8100836)
Supplement: Supplementary file 1 [file nanomaterials-08-00836-s001.pdf]

Supporting Information

Ultra-thin Platinum Deposits by Surface-limited Redox Replacement of Tellurium

F. Haidar[1, 3], M. Maas, A. Piarristeguy [1], A. Pradel [1], S. Cavaliere [3], M-C Record [1,2]

1 Institute Charles Gerhardt of Montpellier, UMR CNRS 5253, Chalcogenide materials and Glasses, University of Montpellier, F-34095 Montpellier Cedex 5, France.

2 Aix-Marseille Univ, Université de Toulon, CNRS, IM2NP, av. Normandie-Niemen, F-13013 Marseille, France

3Institute Charles Gerhardt of Montpellier, UMR CNRS 5253, Aggregates Interfaces and Materials for Energy, University of Montpellier, F-34095 Montpellier Cedex 5, France.

m-c.record@univ-amu.fr

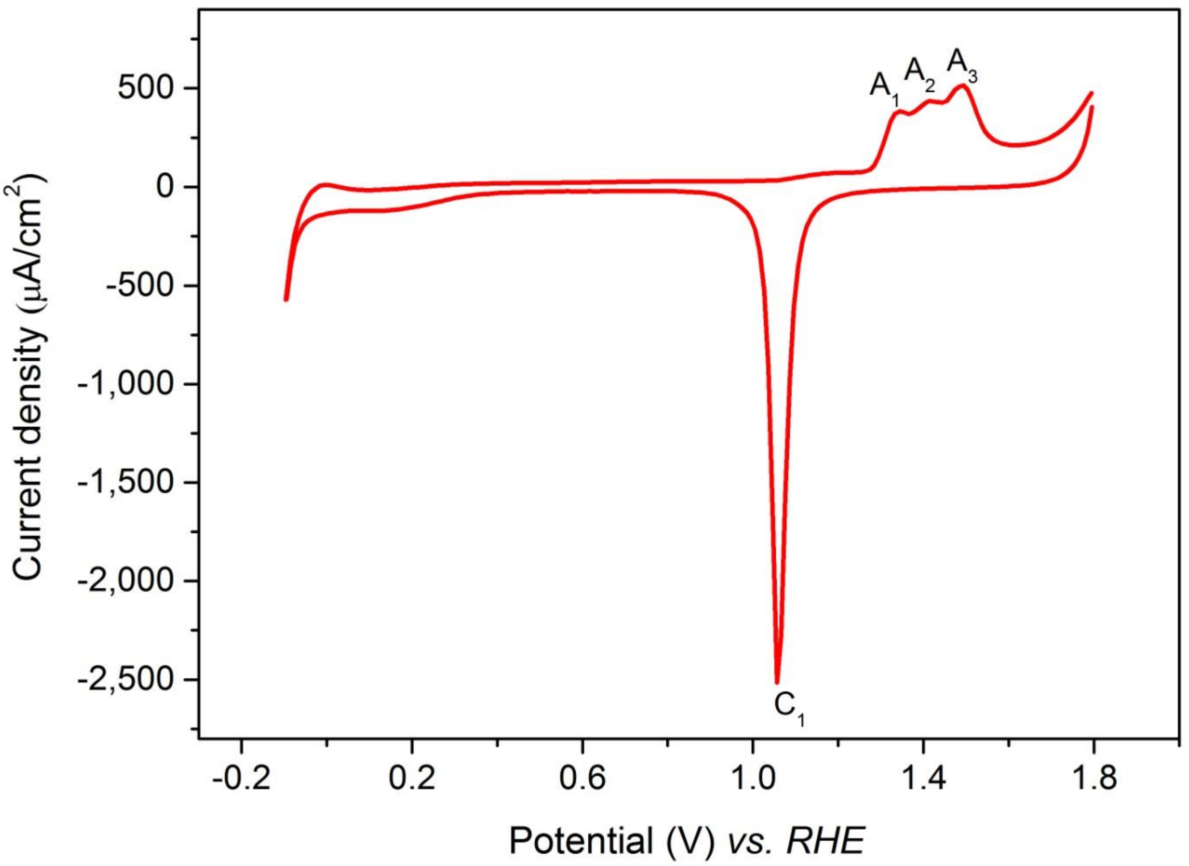

Figure S1: Cyclic voltammogram of the Au electrode in 0.1 M $HClO_4$ at a scan rate of 100 mV/s.

Figure S1 presents the cyclic voltammogram of the gold electrode used for UPD Te deposition. In the anodic branch, A_1, A_2, and A_3 peaks correspond to the oxidation of the surface Au atoms relating to different crystallographic planes, indicating that the electrode is polycrystalline[1]. The C_1 peak, in the cathodic branch, corresponds to the reduction of gold oxides to Au.

a)

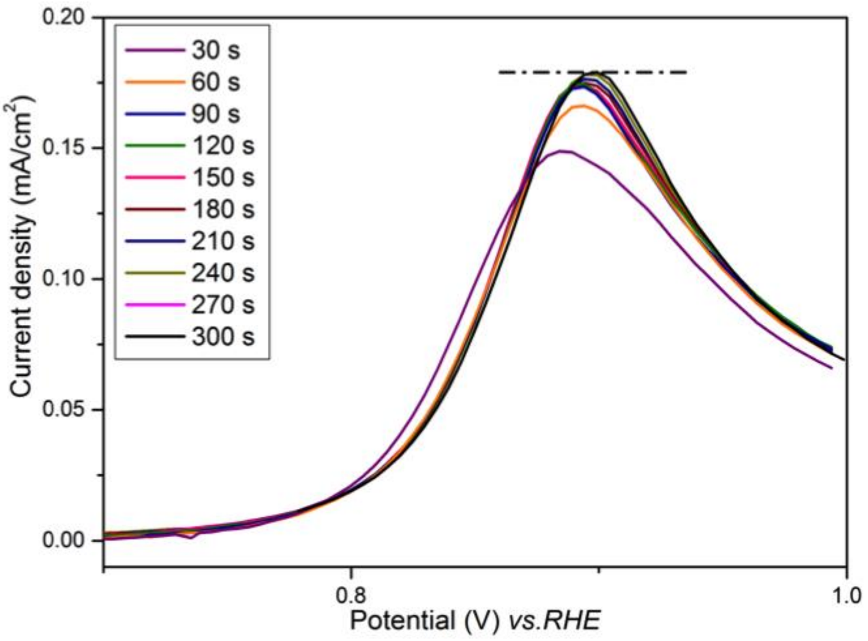

b)

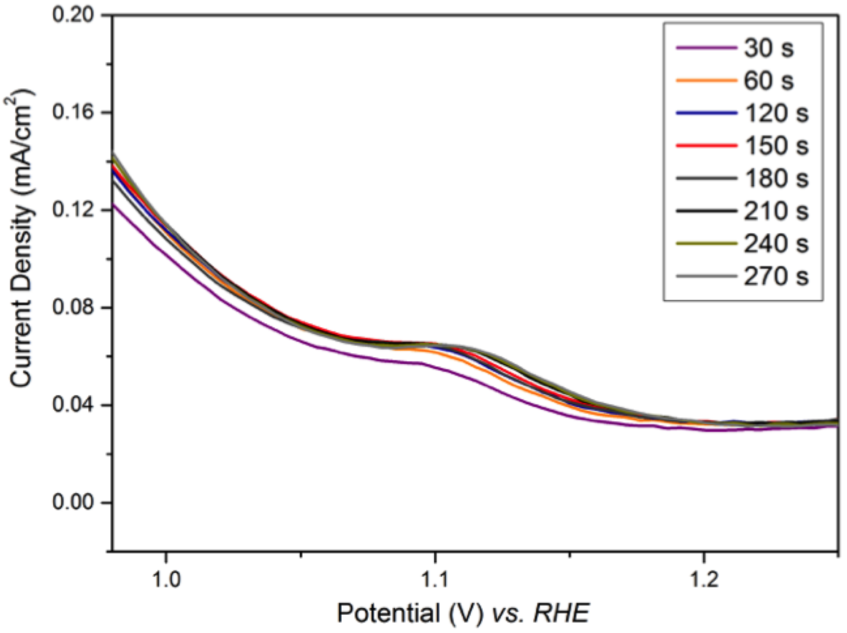

Figure S2. Linear sweep voltammetry after tellurium UPD deposition for various times of deposition at 0.35 V vs RHE. a) peak A_1; b) peak A_2.

The deposition time (Figure S2) was optimized from linear sweep voltammetry (LSV) experiments after UPD Te deposition after polarization at 0.35 V vs RHE for different durations. 240 s was found the minimum time required to achieve a complete UPD. Beyond this duration, the stripping curves overlaid, indicating that the maximum intensity of the stripping peak has been reached.

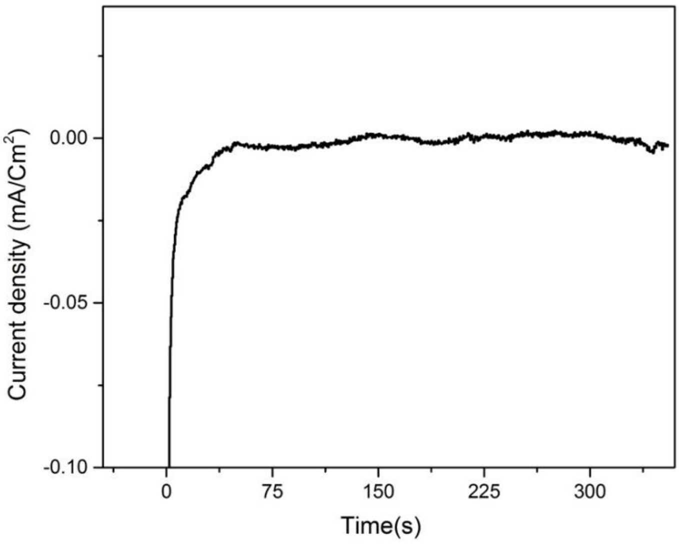

Figure S3. I-t curve during potentiostatic deposition of tellurium at 0.35V.

The [I-t] curve shown in figure S3 was recorded during the deposition at 0.35 V vs RHE. It shows that the signal was stabilized after 240 s

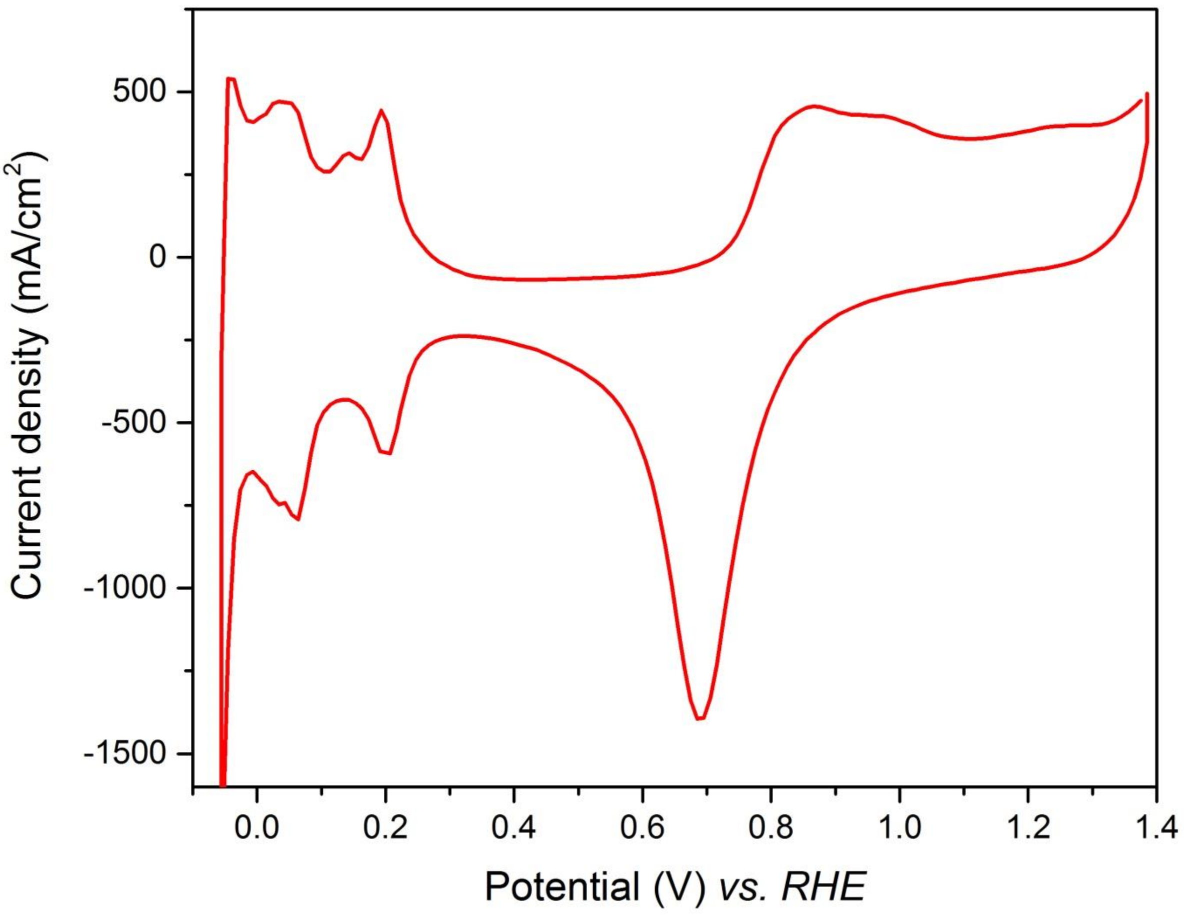

Figure S4. Cyclic voltammogram of the Pt electrode in 0.1 M $HClO_4$ at a scan rate of 100 mV/s.

The voltammogram in Figure S4 recorded on a platinum electrode presents the characteristic hydrogen adsorption/desorption peaks in the low potential region and Pt oxide formation and reduction in the high potential region[2].

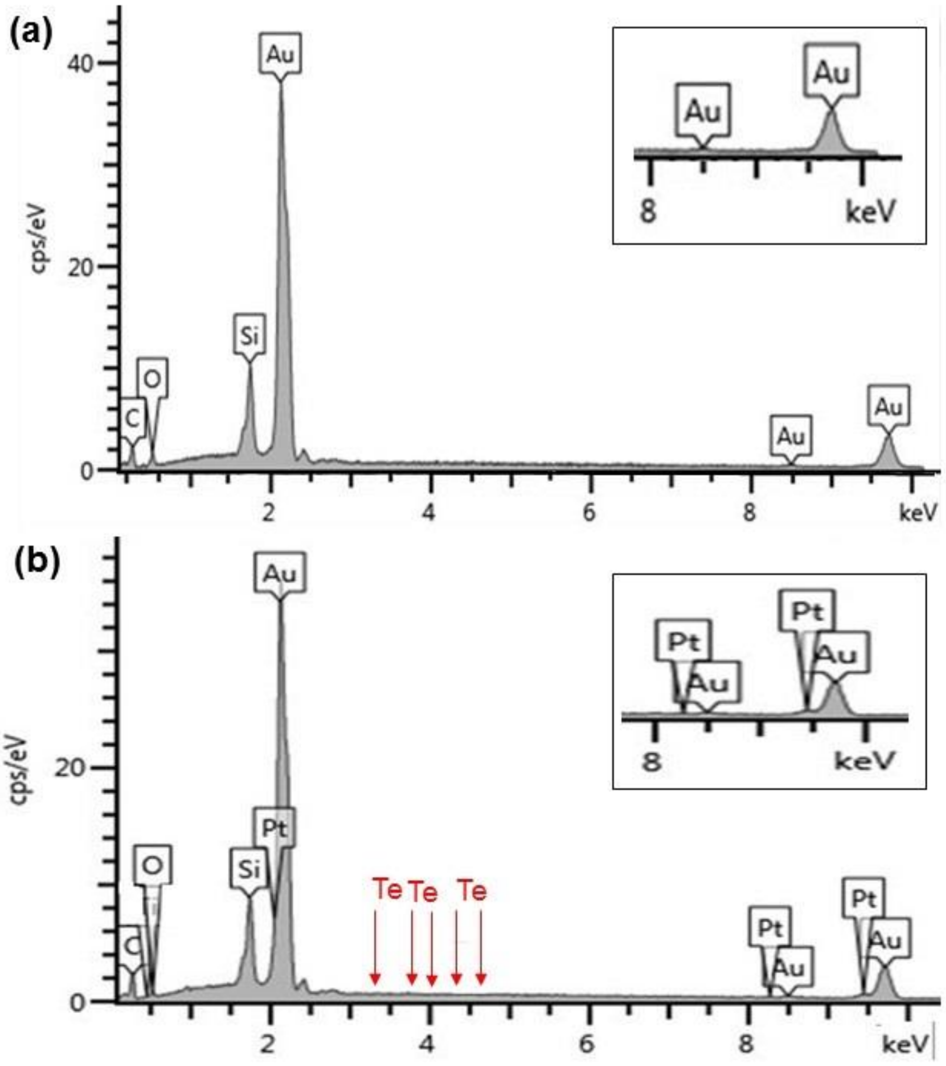

Figure S5. EDS spectra of (a) bare Au-coated glass slide and (b) Pt deposit on Au-coated glass slide. Red arrows indicate the expected position of Te peaks, showing the absence of the element in the layer.

Owing to the low amount of deposited Pt and strong overlap of the main peaks of Au and Pt at 2-2.2 keV, the presence of Pt is to be looked for at the 9.5 keV peak absent in the bare substrate spectrum (a) and present in the Pt covered one (b) (see inset).

1. Stickney, J. L.; Rosasco, S. D.; Schardt, B. C.; Hubbard, A. T. Electrodeposition of silver onto platinum(100) surfaces containing iodineadlattices. Studies by low-energy electron diffraction, Auger spectroscopy, and thermal desorption. *J. Phys. Chem.* **1984**, *88*, 251–258, doi:10.1021/j150646a018.

2. Jerkiewicz, G.; Vatankhah, G.; Lessard, J.; Soriaga, M. P.; Park, Y. Surface-oxide growth at platinum electrodes in aqueous H 2 SO 4 Reexamination of its mechanism through combined cyclic-voltammetry , electrochemical quartz-crystal nanobalance , and Auger electron spectroscopy measurements ℭ. **2004**, *49*, 1451–1459, doi:10.1016/j.electacta.2003.11.008.
